# Alterations in the Myokine Concentrations in Relation to Sarcopenia and Sleep Disturbances: A Narrative Review

**DOI:** 10.3390/jcm14186527

**Published:** 2025-09-17

**Authors:** Michalina Knapik, Jakub Kuna, Grzegorz Chmielewski, Łukasz Jaśkiewicz, Magdalena Krajewska-Włodarczyk

**Affiliations:** 1 Department of Rheumatology, School of Medicine, Collegium Medicum, University of Warmia and Mazury in Olsztyn, 10-719 Olsztyn, Poland; kuna.jakub@wp.pl (J.K.); gchmielewski.gc@gmail.com (G.C.); 2 Department of Human Physiology and Pathophysiology, School of Medicine, Collegium Medicum, University of Warmia and Mazury in Olsztyn, 10-719 Olsztyn, Poland; lukasz.jaskiewicz@uwm.edu.pl

**Keywords:** sarcopenia, sleep disturbances, myokines, irisin, FGF21, BDNF, meteorin, interleukins

## Abstract

**Objectives**: In this study, our aim is to summarise the available data on the correlation between sarcopenia and sleep disturbances as a consequence of changes in the myokine concentrations. **Methods**: Our research was conducted by searching through PubMed, Mendeley and Google Scholar. In our analysis, 63 studies are included from the years 2011 to 2025. Among these studies, there are clinical trials, cross-sectional studies, reviews, systematic reviews and meta-analyses. **Discussion**: There is vast evidence confirming that sleep disturbances are more common among sarcopenic patients. On the other hand, sarcopenia is frequently observed among people with worse quality of sleep. It is also well documented that sarcopenia leads to changes in the myokine serum concentrations, and similar changes are observed among people suffering from sleep disturbances. Sarcopenic patients have lower levels of irisin, BDNF (brain-derived neurotrophic factor), meteorin and IL-15 (interleukin 15) and higher concentrations of FGF-21 (fibroblast growth factor 21) and interleukins 1β, 6 and 10. Lower levels of irisin, BDNF and meteorin, and higher levels of FGF-21 and interleukins 6 and 10, lead to sleep disturbances, like insomnia, reduction of REM (rapid eye movement) sleep time and lower slow-wave activity during the NREM (non-rapid eye movement) sleep phase. These changes are also observed in obstructive sleep apnea (OSA). More severe OSA is correlated with lower levels of irisin and meteorin and higher levels of FGF-21 and interleukins 6 and 8. **Conclusions**: Taking into account the similarities in the myokine concentration changes in sarcopenia and in sleep disturbances, it may be concluded that alterations in the myokine levels induced by sarcopenia provoke sleep disturbances. However, it is necessary to further investigate these correlations to understand them better.

## 1. Introduction

Sarcopenia is a generalised and progressive disorder of the muscle tissue, the main symptom of which is loss of muscle strength [1]. It relates to ageing and is common among the geriatric population. The global prevalence of sarcopenia among individuals over 60 years old varies between 10 and 27% and is constantly increasing due to the ageing of the population in developed countries [2]. Sarcopenia often leads to falls, fractures and physical disability, resulting in a loss of independence and a deterioration in the quality of life of older people [3]. Since 2016, it has been recognised as a geriatric syndrome and classified as a disease in the International Classification of Diseases (ICD-10) [4].

In addition to the primary age-related sarcopenia, one can also distinguish secondary sarcopenia, which can be related to a lack of activity, malnutrition, diseases (e.g., osteoporosis, rheumatoid arthritis, insulin resistance, liver diseases) and drugs (e.g., statins, sulfonylureas, SGLT2 (sodium-glucose cotransporter 2), inhibitors, steroids, chloroquine, colchicine, nucleoside analogues, loop diuretics, androgen deprivation therapy, D-penicillamine, antineoplastic drugs, immune checkpoint inhibitors) [5,6,7].

The pathophysiology of sarcopenia is complex. There are several mechanisms involved in the development of this disease. The principal cause of sarcopenia is the atrophy of individual myofibres and a decrease in their number, which is the result of the denervation of motor units [1]. Ageing causes changes in the neuromuscular synapses, which leads to their impairment [3]. Degeneration of the myofibres is also triggered by oxidative stress and mitochondrial dysfunction [6]. In younger individuals, loss of muscle fibres can be reversible due to satellite cells, but in older people, these cells lose their function, leading to the decreased regenerative capacity of the myofibres [8,9,10]. Hormonal changes occurring in older people also contribute to the development of sarcopenia. An increase in the production of catabolic agents and a decrease in anabolic hormones impair the synthesis of proteins by the muscles [3]. The common chronic inflammation in the geriatric population, due to impairment of the immune system, also favours the degradation of skeletal muscles because of the constantly elevated blood levels of pro-inflammatory cytokines [11]. Myokines also play a significant role in the pathophysiology of sarcopenia. These are biologically active molecules produced by muscle cells. They are released from myofibres and can act paracrinologically and endocrinologically throughout the body [1,12]. Sarcopenia leads to alteration of the myokine blood concentration, which can result in various consequences for the organism, such as increased risk of cardiovascular diseases and cognitive impairment [12,13,14].

Sleep disturbances are common problems among the geriatric population, as well as sarcopenia, which is why researchers have started to look for a connection between these two afflictions. The available studies show bidirectional relationship between sarcopenia and sleep disturbances [15,16,17,18,19,20,21,22,23,24,25,26]. Too long and too short sleeping times enhance the risk of developing sarcopenia [16,17,18,19]. Low sleep quality leads to reduced physical activity, which plays the key role in preventing the development of sarcopenia [1,12]. Age-related sleep changes provoke hormonal imbalance by decreasing secretion of somatotropin, IGF-1 and testosterone and increasing secretion of cortisol and myostatin. It also leads to increased insulin resistance [27]. This hormonal imbalance stimulates muscle catabolism and inhibits anabolism, which leads to sarcopenia [27]. Low sleep quality, as well as sarcopenia, has a significant impact on the quality of life of older adults and correlates with an increased risk of mental and physical health problems [28].

In this study, our aim is to summarise the available data on the correlation between sarcopenia and sleep disturbances as a consequence of changes in the myokine concentrations. We would like to investigate if changes in myokine secretion related to sarcopenia have an influence on sleep quality. An equally interesting issue is the impact of sleep disorders on myokine secretion and, consequently, the development of sarcopenia.

## 2. Materials and Methods

Our research was conducted by searching through PubMed, Mendeley and Google Scholar. We searched for relevant studies by using the following keywords: “sarcopenia”, “sleep disturbances”, “sleep disturbances in sarcopenia”, “myokines and sleep disturbances”, “myokines and sarcopenia”, “irisin and sarcopenia”, “irisin and sleep disturbances”, “BDNF” (brain-derived neurotrophic factor), “IL-6” (interleukin 6), “IL-8” (interleukin 8), “IL-15” (interleukin 15), “FGF-21” (fibroblast growth factor 21), and “meteorin”.

The research was conducted by one researcher between February and June 2025 and approved by the rest of the authors. The research for each section of the study was performed separately.

In our analysis, studies from March 2011 to February 2025 were taken into account. We included 63 studies in total. There were 12 studies on the correlations between sarcopenia and sleep disturbances, 13 on irisin, 12 on brain-derived neurotrophic factor (BDNF), 3 on meteorin, 12 on FGF-21, and 11 on interleukins. Among these studies, there were clinical trials, cross-sectional studies, longitudinal studies, reviews, systematic reviews and meta-analyses. We did not use strict inclusion criteria because of the limited data corresponding to the subject of our study. We excluded only studies older than 2011 and those that are not available in full-text form. We did not perform any quality assessment of the included studies. Table 1 sums up the characteristics and results of the main original studies included in our review.

As our study contains only information from the literature review without extracting personal information, and as we did not perform any experiments on animals or humans, our protocol did not require approval by an bioethics committee or institutional review board.

## 3. Sarcopenia and Sleep Disturbances Correlations

In recent years, many scientists have investigated the correlations between sarcopenia and sleep quality. The results of these studies, as reported in the available literature, clearly indicate the existence of a bidirectional relationship between sarcopenia and sleep disorders [15,16,17,18,19,20,21,22,23,24,25,26]. On the one hand, people with sarcopenia are more likely to suffer from sleep disorders, and on the other, sleep disorders contribute to the development of sarcopenia. The study from 2024 shows statistically significant bidirectional correlations between certain sleep traits and sarcopenia traits [15]. The authors demonstrated that daytime naps lead to a larger waist circumference (OR (odds ratio) 1.234, 95% CI (confidence interval) 1.081–1.408, *p (probability)* = 0.002) and lower walking pace (OR 0.879, 95% CI 0.834–0.928, *p* = 2.289 × 10^−6^). Insomnia is also connected to a lower walking pace (OR 0.871, 95% CI 0.798–0.951, *p* = 0.002) and grip strength in both hands (right hand: OR 0.844, 95% CI 0.747–0.954, *p* = 0.007, left hand: OR 0.836, 95% CI 0.742–0.943, *p* = 0.003). On the other hand, lower appendicular lean mass predisposes individuals to insomnia (OR 0.979, 95% CI 0.969–0.989, *p* = 5.888 × 10^−5^). Worse grip strength is correlated with morning getting up (right hand: OR 1.044, 95% CI 1.001–1.089, *p* = 0.043, left hand: OR 1.048, 95% CI 1.001–1.097, *p* = 0.043) and insomnia (right hand: OR 0.950, 95% CI 0.918–0.983, *p* = 0.003, left hand: OR 0.927, 95% CI 0.893–0.962, *p* = 5.650 × 10^−5^). A larger waist circumference leads to a shortening of sleep time (OR 0.957, 95% CI 0.936–0.979, *p* = 1.747 × 10^−4^), excessive daytime napping (OR 1.060, 95% CI 1.039–1.081, *p* = 1.654 × 10^−8^), and insomnia (OR 1.038, 95% CI 1.017–1.060, *p* = 3.261 × 10^−4^) [15].

Lv et al., based on the China Health and Retirement Longitudinal Study (CHARLS), proved that sleeping for less than 6 h per night favours sarcopenia (OR 1.22, 95% CI 1.04 to 1.44) and low handgrip strength (OR 1.27, 95% CI 1.04 to 1.57) [16]. In contrast, Shibuki et al. described the association between sarcopenia and a sleeping time longer than 8 h. An especially strong association was observed between long sleep and low muscle strength (OR 1.77, 95% CI 1.07–2.94) and low physical performance (OR 1.90, 95% CI 1.25–2.88) [17].

There are also two older studies that reported the increased risk of sarcopenia in both short and long sleepers [18,19].

Another interesting aspect is the correlation between sarcopenia and obstructive sleep apnea (OSA). Three studies show that there is a greater risk of sarcopenia among OSA patients (OR 1.5, 95% CI 1.1–2.7) [20], and low muscle strength (OR 1.68, 95% CI 1.07–2.64) [21] and low muscle mass (OR 2.17, 95% CI 1.92–2.45) [21] are also associated with a greater risk of OSA [20,21,22].

One study from 2024 proved that people with rapid eye movement sleep behaviour disorder (RBD) have reduced temporal muscle thickness (11.843 vs. 10.420 mm, *p* = 0.002), which is an indicator of sarcopenia. Unfortunately, this study included only 28 patients with isolated RBD and 30 healthy people in the control group; therefore, it is not representative and should be further investigated in a larger group [23].

Yildrim et al., in their study, described the correlation between sarcopenia and restless leg syndrome (RLS). They found out that the ratio of sarcopenia is higher among patients with RLS (8% in RLS group vs. 2.3% in control group, *p* = 0.047; OR 4.542, 95% CI [1.284, 16.071]) [24].

There is also a documented link between sarcopenia and insomnia. Among Japanese older adults, the percentage of individuals experiencing difficulty initiating and/or maintaining sleep (DIMS) is significantly higher for those with sarcopenia, reaching 70.9% (OR 1.60, 95% CI 1.14–2.25). What is particularly interesting in this study is that the correlation between DIMS and sarcopenia was observed in adults aged 65–70 and 79–98 years, but not in the group aged 71–78 years [25].

Another study included in our analysis shows the association between sarcopenia and excessive daytime sleepiness (EDS). The number of falls (IRR = 1.94, 95% CI 1.42–2.65) and the presence of sarcopenia (OR 2.41, 95% CI 1.41–4.12) are higher among adults with EDS in both the female and male groups [26].

When analysing the correlation between sleep quality and sarcopenia, it is important to be aware of a number of confounding factors that may influence this relationship. The most important confounders are socio-economic status, lifestyle, socio-demographic characteristics and clinical comorbidities. By socio-demographic characteristics, we mean factors such as age, gender, marital status, educational level and residence type (rural or urban). In terms of comorbidities, the greatest impact on the sleep–sarcopenia relationship is exerted by diseases such as anaemia, depression, cardiovascular diseases, malnutrition, obesity, hypertension, dyslipidaemia, diabetes, chronic diseases of lungs, kidneys and liver, and cancers. The most important lifestyle factors are smoking and alcohol intake [16,17,20,24].

As outlined above, there is ample evidence linking sleep disorders and sarcopenia. However, in this review, we aim to explore, on the basis of the available literature, whether this relationship may be mediated by changes in the myokine concentrations.

## 4. Roles of Selected Myokines in Sarcopenia and Sleep Disturbances

### 4.1. Irisin

Irisin is part of the fibronectin type III domain-containing 5 protein (FNDC5). It is activated by physical activity. Its ratios vary under the influence of various medical conditions. Levels of irisin are higher among oncologic patients and lower in diseases like Alzheimer’s disease, cardiovascular diseases, osteoporosis, muscle atrophy, and obesity [47]. There is also a documented influence of age and gender on irisin levels. According to the Löffler et al.’s study from 2015, the irisin concentration significantly decreases with age and is lower among women than man. However, in children, they observed higher irisin levels among lean girls than boys [48].

Regarding the relationship between irisin and sarcopenia, there are a number of studies confirming lower irisin concentrations in people with sarcopenia [49]. The meta-analysis from 2024 is based on twelve studies, which included 2133 participants. The authors found significantly lower levels of irisin among patients with sarcopenia. They found a positive correlation between the levels of circulating irisin and the muscle mass and strength [50]. Park et al. showed a positive correlation between the irisin levels and the quadriceps cross-sectional area and liver attenuation [29]. Another study, by Chang et al., showed the correlation between the irisin levels and the appendicular lean mass and handgrip strength in both sexes [30]. Those with lower appendicular lean mass and handgrip strength had lower irisin levels. We also found two studies describing the association between irisin and sarcopenia in patients suffering from other chronic diseases, such as type 2 diabetes and liver cirrhosis. In both cases, the irisin concentrations were lower in sarcopenic patients [51,52]. In opposition to the evidence presented above, Baek et al. did not find a correlation between the irisin levels and sarcopenia, but they admitted that they had some limitations. First of all, the lack of a clear definition of sarcopenia and, secondly, the variability in terms of the BMI among the compared patient groups [53].

Changes in the circulating irisin levels are also linked with sleep disturbances. Gamal et al., in their study, proved the correlation between the serum irisin levels and poor sleep quality in rheumatoid arthritis (RA) patients. It was not a big study, because it included only 58 RA patients and 30 healthy controls. Nevertheless, they found that 45% of RA patients suffered from poor sleep quality, and they had significantly lower irisin levels than those without sleep disturbances and healthy controls [40]. There are also several studies about the influence of changes in the irisin levels on the presence and severity of obstructive sleep apnea (OSA). Four independent research groups, which examined a total of 393 OSA patients and 197 healthy controls, determined that OSA patients have lower irisin levels than healthy individuals. Among OSA patients, there were significantly lower irisin levels in severe OSA compared to moderate and mild [41,54,55,56].

The mechanisms underlying the correlation between irisin alterations and linking sarcopenia with sleep disturbances are not clear, but there are some hypotheses. First of all, irisin takes part in the inflammatory processes by suppressing secretion of pro-inflammatory agents like IL-6, NF-kß (Nuclear Factor kappa B), TNF-α (Tumor Necrosis Factor-alpha) and others from adipocytes and macrophages. It is also involved in the recruitment of macrophages and T lymphocytes and enhances endothelial cell integrity [51]. Taking into account this information, it can be concluded that lower levels of irisin predispose individuals to development of inflammation, which favours sleep disturbances [57]. Secondly, irisin plays a role in reducing the risk of cardiovascular diseases, especially cardiac hypertrophy and atherosclerosis [27]. Cardiovascular diseases are well-known risk factor for a deterioration of sleep quality. Respiratory problems resulting from heart failure predispose individuals to frequent awakenings during night sleep and reduced time of the REM sleep phase, which results in extensive daytime sleepiness [58]. Finally, irisin can impact the quality of sleep by regulating BDNF expression. Irisin stimulates BDNF secretion; therefore, low levels of irisin result in low levels of BDNF, and that can impact slow-wave activity during the NREM sleep phase, resulting in worse relaxation and regeneration during sleep [59,60,61].

### 4.2. Brain-Derived Neurotrophic Factor (BDNF)

BDNF is a myokine and neurotrophin that plays a key role in neurodevelopment during childhood and in synaptic transmission and activity-dependent neuroplasticity in the adult brain [62]. A lower concentration of BDNF in older people increases the probability of developing dementia and a decline in cognitive function [13,14]. Several studies indicate the correlation between the BDNF serum levels and sleep disturbances. Higher BDNF increases slow-wave activity during the NREM sleep phase, which allows for better relaxation, regeneration and consolidation of memory traces [59,60,61]. Mikoteit et al. described in their study that patients suffering from insomnia have significantly lower BDNF levels than healthy controls [42]. The study on mice from 2022 confirmed that inhibition of Neurotrophin Tyrosine Kinase Receptor B, which is normally activated by BDNF, results in a reduction in the REM sleep time [63].

There is also evidence of a link between the BDNF serum levels and sarcopenia. Five independent studies indicate that the BDNF serum levels are significantly lower among patients with sarcopenia and frailty [31,64,65,66,67]. However, Pratt et al., in their study, observed elevated concentrations of BDNF in people with sarcopenia [68]. The main difference between these studies is that in those where the BDNF levels were lower in sarcopenic patients, the patients suffered from other diseases than sarcopenia and had poorer cognitive status, whereas in Pratt et al.’s study, the patients suffered only from sarcopenia and did not have problems with cognitive function. On the other hand, the mean age of the participants in Pratt et al.’s study was around 64 years, while in those studies with lower BDNF, the participants were also older.

BDNF supports skeletal muscle integrity by stabilising neuromuscular junctions. It also participates in the regulation of the proliferation and differentiation of satellite cells and supports glucose metabolism by stimulating glucose transport and increasing insulin sensitivity [68]. All these mechanisms support skeletal muscle regeneration; therefore, lower BDNF levels favour sarcopenia. Regarding the connection between BDNF and sleep disturbances, there is evidence that BDNF takes part in the regulation of slow-wave activity during sleep by stimulation of tyrosine kinase B receptor and cAMP-response element-binding protein [61].

### 4.3. Meteorin-like Protein (Metrnl)

Meteorin-like protein is secreted in skeletal muscles and adipose tissue after physical activity and exposure to cold. It regulates the browning of the white adipose tissue and improves glucose tolerance. It also plays a role in the regeneration of skeletal muscles by an anti-inflammatory effect [69].

The study from 2024 conducted by Wang et al. proved that sarcopenia leads to lower serum levels of meteorin-like protein. They examined 772 older adults, of whom 499 had diagnosed sarcopenia and 323 were healthy. For the assessment of sarcopenia, they measured the gait speed, appendicular skeletal muscle mass index (ASMI) and handgrip strength. The study showed a positive correlation between the Metrnl serum concentration and all the components of sarcopenia. Patients with sarcopenia had significantly lower Metrnl serum levels than healthy controls [32].

A lower serum concentration of the meteorin-like protein is also observed in obstructive sleep apnoea. The study conducted by Sun et al. included 207 patients with obstructive sleep apnoea and 106 healthy individuals. OSA patients had significantly lower serum concentrations of the meteorin-like protein than healthy controls, and among the OSA patients, a higher severity of disease was associated with lower concentrations of meteorin [43].

The influence of meteorin serum levels alterations on developing sarcopenia and sleep disturbances may be related to meteorin’s impact on inflammatory mechanisms. Metrnl induces IL-4/IL-13, which activates anti-inflammatory and inhibits pro-inflammatory cytokines [32,43]. A low meteorin concentration favours inflammation, which is a risk factor for sarcopenia as well as sleep disturbances [57]. Secondly, meteorin plays a role in glucose metabolism in muscle tissue. A low meteorin level enhances insulin resistance and protein degradation and inhibits protein synthesis, which leads to sarcopenia [32]. However, there is not enough data available on this topic and these mechanisms and the link between meteorin and sarcopenia and sleep disturbances need further investigation.

### 4.4. FGF-21

Fibroblast growth factor 21 is part of the FGF family, produced mainly by the liver but also the pancreas, skeletal muscles and white adipose tissue. It takes part in the modulation of cell metabolism, growth, differentiation and proliferation. It also stimulates the uptake of glucose and plays a role in gluconeogenesis and ketogenesis. The secretion of FGF-21 is stimulated by starvation and physical activity [70,71].

Regarding the relationship between FGF-21 and sarcopenia, two independent studies indicate higher FGF-21 serum levels among sarcopenic patients than healthy controls. Both included people above 65 years old with primary sarcopenia and both revealed an inverse correlation of the FGF-21 level with handgrip strength. A Korean study also showed an inverse correlation with the skeletal muscle mass index and a positive correlation with the sarcopenia phenotype score, while a Turkish study revealed a positive correlation with the 6 min walk test. A total of 213 patients were examined in these two studies, including 64 diagnosed with sarcopenia [33,72]. However, the meta-analysis from 2023 indicates a lack of strong evidence for the relationship between increased FGF-21 serum levels and sarcopenia [73]. This analysis includes five studies—two of which are described above and three others. These three studies differ from those described above because they included secondary sarcopenia patients. One of them concerns only female patients with diabetes mellitus, and the second one newly concerns diagnosed colorectal cancer patients [74,75]. The last one examined not only the FGF-21 levels but also other myokines. None of these three showed a positive correlation between high FGF-21 serum levels and sarcopenia [76]. Zhou et al. in 2024 carried out a study that included mouse models and patients with decompensated cirrhosis (DC) [77]. They examined the correlation between the FGF-21 serum levels and DC-related sarcopenia. The study showed significantly elevated serum levels of FGF-21 in DC-related sarcopenia. Liver-secreted FGF-21 inhibits satellite cells’ function, which leads to sarcopenia [77].

In regard to the relationship between the FGF-21 concentrations and sleep disorders, we found three studies. The first one explored the impact of acute sleep loss on the FGF-21 serum levels. It indicated that acute sleep loss increases the FGF-21 serum concentrations in healthy young men [78]. The second study described the correlation between the FGF-21 levels and obstructive sleep apnea. OSA patients have significantly higher levels of FGF-21 than healthy individuals, and this increases in parallel with increasing OSA severity [79]. The third study focused on the relation of sleep quality to fourteen cardiometabolic markers (among them FGF-21) in airline pilots. It included 117 airline pilots, of whom 47 suffered from poor sleep quality. The study demonstrated that sleep disturbances among airline pilots are correlated with significantly higher levels of FGF-21 and GDF-15 as well as lower levels of adiponectin [44].

The mechanisms underlying FGF-21’s connection to sarcopenia and sleep disturbances are still not well understood. Nevertheless, it is known that FGF-21 impairs the function of satellite cells by alteration of the PI3K/Akt pathway, which leads to worse muscle fibre regeneration and, in consequence, to sarcopenia [77]. FGF-21 also has a catabolic effect on muscle tissue [33]. In regard to sleep disturbances, there is evidence of FGF-21’s influence on regulation of the circadian rhythm by engaging hypothalamic pathways. However, it was explored only in an animal model [80].

### 4.5. Interleukins

Interleukins are proteins belonging to the cytokine family and they play a key role in the regulation of immune processes and take part in haematopoiesis. They allow mutual interactions of different leukocyte groups. They can be divided into pro-inflammatory and anti-inflammatory interleukins. Some of them take part in the pathophysiology of sarcopenia as myokines [70,81].

The meta-analysis from 2024 showed correlations between different interleukins and sarcopenia. Its authors analysed 37 studies in total, including 2978 sarcopenia patients and 5016 healthy controls. They examined seven interleukins: IL-1β, IL-4, IL-6, IL-8, IL-10, IL-12, and IL-17. The study revealed significantly higher levels of IL-1β, IL-6 and IL-10 in patients with sarcopenia and no significant association between IL-4, IL-8, IL-12 and IL-17 and sarcopenia. However, there was a smaller sample of studies about interleukins 4, 8, 12 and 17 than 1β, 6 and 10 [82]. As for interleukin 8, there is a study, not included in the aforementioned meta-analysis, which showed a correlation between elevated IL-8 levels and sarcopenia. However, it relates to cancer-related sarcopenia in pancreatic cancer patients [83]. There are also two studies indicating lower IL-15 levels among sarcopenic patients [34,84].

When it comes to the relationship between interleukins and sleep disorders, there is evidence of a correlation between IL-6, IL-8 and IL-10 and sleep quality. High levels of IL-6 are correlated with sleep disturbances, especially with extremely long sleep durations [57]. Older adults with worse sleep quality often have chronically high levels of IL-6 [45]. Sleep deprivation provokes an increase in the IL-6 levels the following day, which leads to enhanced sleepiness during the day and daytime napping [85]. There is also evidence of increased IL-6 and IL-8 levels among obstructive sleep apnea (OSA) patients [46]. Additionally, the IL-8 concentration is higher in severe OSA than in mild and moderate [86]. Poor sleep quality is also significantly positively correlated with the IL-10 concentration [87].

It is well known that the ageing process is accompanied by chronic low-grade inflammation, which leads to an increased incidence of various diseases such as hypertension, dyslipidaemia, cardiovascular events, diabetes type 2 and obesity. It also promotes autoimmunologic diseases [57,88]. Chronic inflammation is associated with elevated levels of pro-inflammatory cytokines, which affect various physiological processes, including, among others, the sleeping pattern and muscle metabolism [57,88]. Higher levels of CRP and IL-6 intensify muscle strength loss [70]. Sleep deprivation and inflammation are also correlated bidirectionally. Inflammation affects sleep quality and sleep deprivation accelerates inflammation [45,46,57,85,86,87].

## 5. Discussion

Sarcopenia and sleep disturbances are correlated with each other bidirectionally. Except for the role of the myokines in this relationship, which was broadly discussed in this article, there are other possible mechanisms. Sleep deprivation provokes hormonal changes, which lead to metabolic imbalance. Lower secretion of somatotropin, IGF-1 and testosterone and higher secretion of cortisol and myostatin, as well as greater insulin resistance, enhance muscle catabolism and inhibit anabolism. This imbalance between muscle catabolism and anabolism favours sarcopenia [27,89,90].

**Figure 1:**
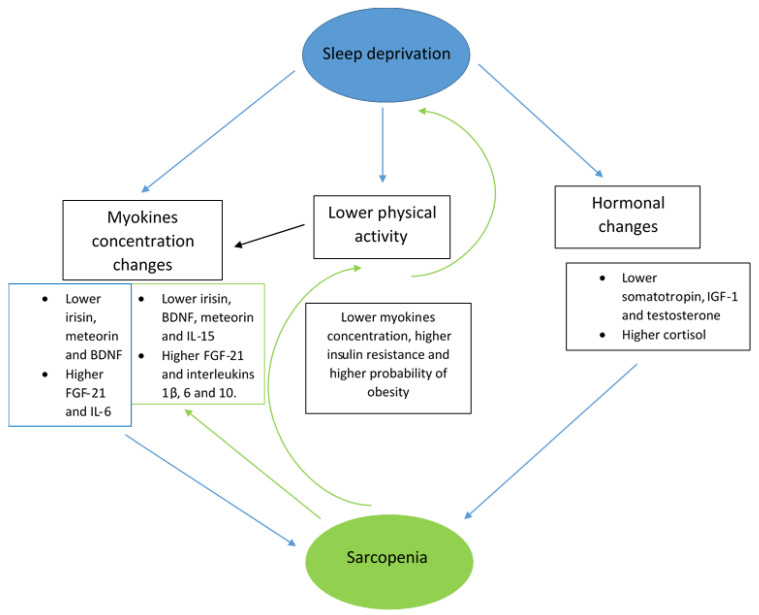
Bidirectional correlations between sleep deprivation and sarcopenia. Abbreviations: BDNF—brain-derived neurotrophic factor, FGF-21—fibroblast growth factor 21, IL-6—interleukin 6, IL-15—interleukin 15, IL-1β—interleukin 1β, IGF-1—insulin-like growth factor 1. Arrows indicate the directions of the correlations between sarcopenia and sleep disturbances.

Another key factor connecting sarcopenia with sleep impairment is physical activity. It plays a crucial role in preventing sarcopenia and ensuring good sleep quality [12,27]. Especially, aerobic exercises improve sleep quality and reduce depression and anxiety [27]. Physical activity regulates protein and lipid metabolism. It also has a positive influence on the equilibrium between pro- and anti-inflammatory mediators [12]. People who exercise regularly are less likely to be obese, and obesity promotes sarcopenia and sleep disturbances [90]. There is evidence that physical activity enhances secretion of myokines such as irisin, BDNF, FGF-21, meteorin and IL-6 [13,14,27,48,65,67]. On the other hand, poor sleep quality leads to decreased physical activity, which favours sarcopenia. Sarcopenia also impairs physical activity, which may lead to sleep deprivation. Figure 1 shows the aforementioned relationships between sleep quality and sarcopenia.

Given the increasing recognition of the interrelated nature of musculoskeletal decline, sleep regulation, and molecular signalling pathways, there is a growing need for integrative articles that address the longitudinal associations among sarcopenia, sleep disturbances, and myokines. Although sarcopenia and impaired sleep frequently co-occur in older adults, the mechanisms underlying their bidirectional relationship remain insufficiently clarified. Myokines, as muscle-derived cytokines with systemic effects, represent a plausible biological link, given their roles in inflammation, metabolic regulation, and circadian rhythm modulation. Synthesising the evidence across clinical cohorts, mechanistic studies, and translational research is therefore essential to delineate causal pathways, establish early predictive markers, and identify potential therapeutic targets. A comprehensive, longitudinal perspective would provide critical insights into how these domains interact over time and could inform the development of interventions aimed at simultaneously preserving muscle health, improving sleep quality, and optimising myokine signalling.

## 6. Limitations

Our article has several methodological limitations. First of all, it contains information from many studies performed over a period of 14 years, and the studies differ in terms of the nature of the research, but also in terms of the size of the study groups. The inclusion criteria were not very strict, because there are not a lot of studies corresponding to our topic and we tried to include as many as possible to make the article more valuable. Secondly, we did not perform any quality assessment of the included studies. Finally, the research was performed by only one researcher.

Another limitation of this study is the fact that most of the analysed studies are cross-sectional, and only few of them are longitudinal. In addition to that, the included studies analysed separately the connections between myokine levels changes and sleep disturbances and sarcopenia.

## 7. Future Research Directions

Future research on the subject of sarcopenia and sleep correlations and the role of myokines in this relationship should deepen the knowledge about the mechanisms of myokines’ action in the pathophysiology of sarcopenia and sleep deprivation. There is also a need for more longitudinal studies on this topic, as to date most of the studies are cross-sectional. Integrative studies on the longitudinal association between sarcopenia, sleep disturbances, and myokines should be conducted to find potential therapeutic targets. Myokines are a promising therapeutic target for the treatment of sleep disorders and sarcopenia; therefore, it is important to gain a better understanding of these relationships in order to be able to help older people improve their quality of life more effectively.

## 8. Conclusions

As presented in our study, there is vast evidence of a correlation between sarcopenia and sleep disturbances. Studies proved that people with sarcopenia more often have various sleeping problems. Among sarcopenic patients, shortening of the sleep time, excessive daytime napping, and insomnia occur more frequently. On the other hand, lower quality of sleep enhances sarcopenia. Sarcopenia is more frequent among OSA patients and patients with RLS. The increased risk of sarcopenia is observed among people sleeping for less than 6 h and more than 8 h.

There are also many studies proving the changes in the myokine concentrations as a consequence of sarcopenia and the link between alterations in the myokine levels and sleep disturbances. Sarcopenia leads to lower levels of irisin, BDNF, meteorin and IL-15, as well as higher concentrations of FGF-21 and interleukins 1β, 6 and 10. When it comes to interleukins, the relationship is bidirectional. Chronic inflammation in older people leads to chronically elevated levels of interleukins, which induces sarcopenia. Changes in the myokine levels are also connected with sleep disturbances. Lower irisin levels are linked with lower sleep quality among rheumatoid arthritis patients and with obstructive sleep apnea. More severe OSA patients have lower irisin levels. OSA patients also have lower levels of meteorin and higher levels of FGF-21 and interleukins 6 and 8. Lower BDNF levels lead to insomnia, a reduction of REM sleep time and lower slow-wave activity during the NREM sleep phase. Higher FGF-21 levels were observed among airline pilots with sleep disturbances. Higher IL-6 levels predispose individuals to extremely long sleep durations.

Considering the similarities in the myokine concentration changes in sarcopenia and sleep disturbances, it may be concluded that alterations in the myokine levels induced by sarcopenia may provoke sleep disturbances and that sleep disturbances may lead to alterations in the myokine levels connected to sarcopenia. It is clear that the relationships are bidirectional; however, further investigation is necessary to better understand these correlations.

## Figures and Tables

**Table 1:** Characteristics and results of the main original studies included in the review.

No.	Title and Authors	Country	Study Population			Sarcopenia Parameters/ Diagnostic Criteria (1–11)	Main Findings	Study Design
			N	Age	Gender	Sleep Disturbances Parameters (12–18)		
Sarcopenia-related studies								
1.	Park H.S. et al. [29]	South Korea	153 + 147 in control group	60+	Females	Quadriceps cross-sectional area, bone mineral density, liver attenuation in CT; muscle strength and physical performance were evaluated by handgrip test and short physical performance battery, respectively.	Circulating irisin was significantly lower in the sarcopenia group. Positive correlation with quadriceps cross-sectional area and liver attenuation was observed.	Cross-sectional
2.	Chang J.S. et al. [30]	South Korea	715	18–90	Females and males	Skeletal muscle mass defined as the sum of appendicular lean mass from all four limbs and adjusted for height squared in meters. Handgrip strength measured by a dynamometer.	Circulating irisin levels were correlated with appendicular lean mass/height^2^ and handgrip strength in both sexes (all *p* < 0.01). The mean circulating irisin levels were lower in the sarcopenia group than in the control group.	Cross-sectional
3.	Miyazaki S. et al. [31]	Japan	20	65+	Females and males	Handgrip strength, 6 m walk test, Short Physical Performance Battery, 5-times chair stand test	BDNF (brain derived neurotrophic factor) serum levels are significantly lower among patients with sarcopenia and frailty.	Cross-sectional
4.	Wang Z.Y. et al. [32]	China	772	65+	Females and males	Muscle mass measured by bioimpedance analysis; grip strength measured by dynamometer; appendicular skeletal muscle mass index; 6 m walk test	Patients with sarcopenia had significantly lower Metrnl serum levels than healthy controls.	Cross-sectional
5.	Bag Soytas R. et al. [33]	Turkey	88	65+	Females and males	Sarcopenia was determined by handgrip strength (HGS), bioelectrical impedance analysis and 6 m walk test.	Study revealed a positive correlation with the 6 min walk test and inverse correlation with the handgrip strength. Study indicate higher FGF-21 serum levels among sarcopenic patients than healthy controls.	Cross-sectional
6,	Yalcin A. et al. [34]	Turkey	160	65+	Females and males	Skeletal muscle mass was measured by bioelectrical impedance analysis (BIA). Handgrip strength was measured using a handheld digital dynamometer. Muscle function by 4 m gait speed. Sarcopenia was diagnosed according to the EWGSOP (European Working Group on Sarcopenia in Older People) criteria.	Study showed lower IL-15 levels among sarcopenic patients.	Cross-sectional
7.	Harada H. et al. [35]	Japan	132	27–93	Females and males	Sarcopenia was determined by AWGS (Asian Working Group on Sarcopenia) diagnosis criteria.	Serum levels of IL-6 were significantly higher in sarcopenic than in non-sarcopenic patients.	Retrospective
8.	Choi K. et al. [36]	Korea	238	60+	Females and males	Sarcopenia was determined by Psoas Muscle Index.	Serum levels of IL-6 were significantly higher in sarcopenic than in non-sarcopenic patients.	Prospective cohort
9.	Bian A.L. et al. [37]	China	441	60+	Females and males	Sarcopenia was determined by EWGSOP diagnostic criteria.	Serum levels of IL-6 were significantly higher in sarcopenic than in non-sarcopenic patients.	Cross-sectional
10.	Kwak Y. et al. [38]	Korea	96	60+	Females and males	Sarcopenia was determined by AWGS diagnostic criteria.	Serum levels of IL-6 were significantly higher in sarcopenic than in non-sarcopenic patients.	Prospective longitudinal
11.	Kamijo Y. et al. [39]	Japan	119	66+	Females and males	Sarcopenia was determined by AWGS diagnostic criteria.	Serum levels of IL-6 were significantly higher in sarcopenic than in non-sarcopenic patients.	Prospective cohort
Sleep-disturbance-related studies								
12.	Gamal R.M. et al. [40]	Egypt	58 RA patients + 30 in control group	18–71	Females and males	Sleep quality was measured by the Pittsburgh Sleep Quality Index.	Irisin levels were lower in RA patients with poor sleep quality compared to RA patients with good sleep quality and healthy controls.	Cross-sectional
13.	Li Y. et al. [41]	China	165 + 98 in control group	54 ± 6	Males	Obstructive sleep apnea (OSA) diagnosed through polysomnography.	OSA patients have lower irisin levels than healthy individuals. Among OSA patients, there were significantly lower irisin levels in severe OSA compared to moderate and mild.	Cross-sectional
14.	Mikoteit T. et al. [42]	Switzerland	60 + 30 in control group	18–65	Females and males	Total sleep time (TST), sleep efficiency (ratio of TST to time in bed), sleep onset latency (SOL), number of awakenings, wake-time after sleep onset, sleep architecture measures measured from EEG (electroencephalogram) records.	Patients suffering from insomnia have significantly lower BDNF levels than healthy controls.	Case-control
15.	Sun H. et al. [43]	China	207 + 106 in control group	54 ± 6	Males	OSA diagnosed through polysomnography.	OSA patients had significantly lower serum concentration of the meteorin-like protein than healthy controls, and among OSA patients, a higher severity of disease was associated with lower concentrations of meteorin.	Cross-sectional
16.	Liaño Riera M. et al. [44]	Spain	117	41 ± 6	Males	Sleep quality was measured by the Pittsburgh Sleep Quality Index.	The study demonstrated that sleep disturbances among airline pilots are correlated with significantly higher levels of FGF-21 and GDF-15 as well as lower levels of adiponectin.	Cross-sectional
17.	Stahl S.T. et al. [45]	USA	195	60+	Females and males	Sleep quality was measured by the Pittsburgh Sleep Quality Index.	Older adults with worse sleep quality often have chronically high levels of IL-6.	Longitudinal
18.	Fiedorczuk P. et al. [46]	Poland	80	20–65	Females and males	OSA diagnosed through polysomnography.	There are increased IL-6 and IL-8 levels among obstructive sleep apnea (OSA) patients.	Case-control
